# Supplementary material for: High school science fair: What students say—mastery, performance, and self-determination theory
Source: PLoS One. 2025 Jun 25;20(6):e0325283. doi: 10.1371/journal.pone.0325283 (PMC12193632; doi:10.1371/journal.pone.0325283)
Supplement: S1 Table — (PDF) [file pone.0325283.s002.pdf]

S1 Table. Quantitative student responses to the SEF survey year by year. - Questions 1-9

SEF Survey Questions and Answers		% Students	
Questions	Answers	(21-22) 657*	(22-23) 534**
1. What grade are you in?	9	43.3	41.9
	10	30.2	32.0
	11	17.6	20.2
	12	8.7	5.8
2. Location of high school?	Urban	20.8	27.0
	Suburban	72.8	66.9
	Rural	3.6	3.7
2A. Type of high school? (22-23 only)	Public	NA	85.8
	Private	NA	7.1
	Charter	NA	6.2
3. Gender?	Female	52.4	55.4
	Male	45.4	43.4
4. Ethnicity most identified with?	Asian	30.4	29.0
	Black	7.9	9.2
	Hispanic	17.6	19.9
	White	38.9	36.5
	Other*	3.5	3.4
	Specify	-	-
5. During high school have you carried out science fair more than once?	Once	67.3	67.4
	> Once	32.4	31.8
6. In which science fair competitions did you compete this year (could be more than one)?	School	71.7	70.0
	District	32.4	27.0
	Region	24.5	30.3
	State	2.6	2.6
7. Was your science fair project team or individual?	Individual	65.5	61.0
	Team	32.5	34.6
8. Was the science fair project required by your school?	Required	64.9	59.2
	Optional	16.4	21.9
	Project	16.6	14.8
9. Do you think science fair projects should be optional or required?	Optional	72.8	75.7
	Required	26.6	22.7
10. Do you think science fair projects for competition should be optional or required?	Optional	87.4	85.4
	Required	12.2	12.9
11. Who helped you with your science fair project?	Parents	51.5	53.0
	Siblings	14.0	11.0
	Other family members	5.6	6.6
	Teachers	54.9	54.9
	Other students	32.2	30.3
	Scientists	5.9	8.1
	A paid mentor	1.1	0.9
	Articles on the Internet	55.8	53.7
	Articles in books or magazines	18.5	18.0
	Other	3.0	6.6
	Specify:	-	-

S1 Table - Questions 10-13

Questions	Answers	(21-22) 657*	(22-23) 534**
10. Do you think science fair projects for competition should be optional or required?	Optional	87.4	85.4
	Required	12.2	12.9
11. Who helped you with your science fair project?	Parents	51.5	53.0
	Siblings	14.0	11.0
	Other family members	5.6	6.6
	Teachers	54.9	54.9
	Other students	32.2	30.3
	Scientists	5.9	8.1
	A paid mentor	1.1	0.9
	Articles on the Internet	55.8	53.7
	Articles in books or magazines	18.5	18.0
	Other	3.0	6.6
	Specify:	-	-
12. What kind of help did you actually receive?	Being given the main idea	10.9	11.2
	Development of the idea	30.5	28.3
	Gathering background research information, or finding a research site or participants	46.8	41.0
	Performing the experiments	38.8	38.6
	Writing the report	13.8	11.0
	Fine tuning the report after it is written	28.7	29.8
	Designing the poster board and presentation	21.3	21.5
	Producing charts or graphs	17.5	16.3
	Coaching for the interview with judges	9.9	8.8
	Copying the project from someone else	0.8	0.2
	Other	5.9	9.2
	Specify:	-	-
13. Did you get the kind of help you wanted from teachers?	Yes	84.3	80.1
	No	14.1	17.4

S1 Table - Questions 14-17

Questions	Answers	(21-22) 657*	(22-23) 534**
14. Was there some kind of help that you would have liked but did not receive?	Specify:	-	-
15. Did you get the amount of help you wanted from teachers?	Yes	81.5	76.8
	No	16.9	20.2
16. What types of communication and presentation skills did you use in your science fair project?	Written report	51.5	57.9
	Literature review	15.3	19.5
	Research notebook	29.5	32.8
	Poster board preparation	62.2	78.5
	Powerpoint presentation	44.7	27.2
	Software to prepare tables, graphs, or images	46.0	52.1
	Presentation to other students	0.0	42.9
	Interview with the judges	43.0	50.4
	Other	2.4	0.9
	Specify?	-	-
17. What obstacles did you face?	Coming up with the main idea	46.7	43.3
	Getting motivated to do the project	48.8	42.1
	Becoming disappointed with the project	24.0	26.8
	Limited resources	33.1	37.5
	Limited knowledge	26.0	28.5
	Limited skills	18.2	21.3
	Limited cooperation	12.8	11.0
	Getting organized	26.9	25.3
	Time pressure	60.5	59.2
	Not enough money	9.7	11.8
	Results not as expected	24.9	24.7
	Other	4.0	3.9
	Specify?	-	-

S1 Table - Questions 18-20A

Questions	Answers	(21-22) 657*	(22-23) 534**
18. How did you overcome the obstacles you encountered?	Used someone elses main idea	1.5	1.7
	Picked a familiar/interesting topic	34.3	31.1
	Did more background research	48.8	50.2
	Stopped working on the project for a while	19.6	17.0
	Made a timeline to follow	27.1	26.6
	Perseverance and self-discipline	50.8	50.7
	Had someone else to keep me on track	15.0	16.7
	Had someone else do the math	0.3	0.2
	Changed the research plan	18.4	15.7
	Collected more data	28.0	23.6
	Had someone else collect the data	1.4	1.5
	Used someone elses data	0.6	0.2
	Made up the data	1.1	1.3
	Changed the hypothesis to fit the data	3.2	5.6
	Changed the data to fit the hypothesis	1.2	0.2
	Other	5.2	6.7
	Specify?	-	-
19. Are you interested in a career in the sciences or engineering?	Yes	58.1	62.0
	Not sure	27.2	22.7
	No	14.7	14.8
20. Did your science fair experience increase your interest in the sciences or engineering?	Yes	56.1	56.0
	No	43.8	44.0
20A. "Reason why?	-	-	-
*Students who answered 20A "Reason Why?" question (977 students total completed surveys)			
**Students who answered 20A "Reason Why?" question (813 students total completed surveys)			
