# Supplementary material for: High school science fair: What students say—mastery, performance, and self-determination theory
Source: PLoS One. 2025 Jun 25;20(6):e0325283. doi: 10.1371/journal.pone.0325283 (PMC12193632; doi:10.1371/journal.pone.0325283)
Supplement: S2 Table — (PDF) [file pone.0325283.s003.pdf]

S2 Table. # comments/100 students in relationship to student demographics and SEF experiences

Survey Question		# of Comments/100 Student								
SEF participation increased my interest in S&E	Yes	42.4	30.1	21.9	13.8		0.6	0.3	0.1	0.4
	No	2.5	4.4	2.7	4.0		33.5	27.7	22.0	16.4
Interest in a career in S&E	Yes	30.2	23.3	13.6	12.2		8.0	13.5	1.7	4.2
	No	7.4	6.8	8.0	3.4		33.0	5.7	46.0	17.6
	Unsure	22.7	15.0	16.3	6.3		21.0	13.7	7.3	9.3
Science fair requirement	Optional	33.0	27.7	17.4	11.2		2.2	3.6	1.8	0.9
	Required	22.3	16.0	11.7	8.9		18.4	15.1	12.4	10.0
	Project	25.0	21.3	18.1	10.6		12.8	11.7	8.5	5.9
Help from scientists	Yes	28.0	32.9	15.9	19.5		6.1	3.7	3.7	2.4
	No	24.6	17.8	13.3	8.7		15.7	13.0	10.2	7.8
Received coaching for interview	Yes	33.9	30.4	16.1	16.1		4.5	3.6	2.7	0.9
	No	23.9	17.6	13.2	8.8		16.1	13.3	10.5	8.2
Competition level	Beyond school	33.3	24.1	16.7	13.5		5.1	4.7	4.1	1.4
	School only	18.9	15.1	12.7	7.3		21.4	18.1	13.2	12.0
Grade in which students participated in SEFs	9	24.2	17.5	15.1	9.8		14.5	11.8	9.0	6.3
	10	22.0	16.5	10.3	8.1		22.5	14.4	13.0	10.6
	11	28.6	23.7	12.1	9.8		8.9	13.8	4.9	7.1
	12	30.7	23.9	20.5	12.5		2.3	3.4	12.5	2.3
Student ethnicity	Asian	29.9	23.2	13.0	9.0		9.6	9.0	5.9	5.6
	Black	24.8	15.8	11.9	3.0		21.8	10.9	11.9	8.9
	Hispanic	30.2	13.5	14.9	13.1		11.3	6.8	11.3	9.5
	White	19.5	18.4	12.9	10.0		18.6	18.2	12.0	7.3
Selected "Reason Why?" Categories		Learned new things	Experience doing research	Enjoyed/fun experience	Career choice		Not fun/ stressful/ boring	Not a good project	Not interested in science	Required
