# Supplementary figures and images for: High school science fair: What students say—mastery, performance, and self-determination theory
Source: PLoS One. 2025 Jun 25;20(6):e0325283. doi: 10.1371/journal.pone.0325283 (PMC12193632; doi:10.1371/journal.pone.0325283)

S1 Figure. Distribution of students' comments in answer to the *Reason Why?* question year by year.

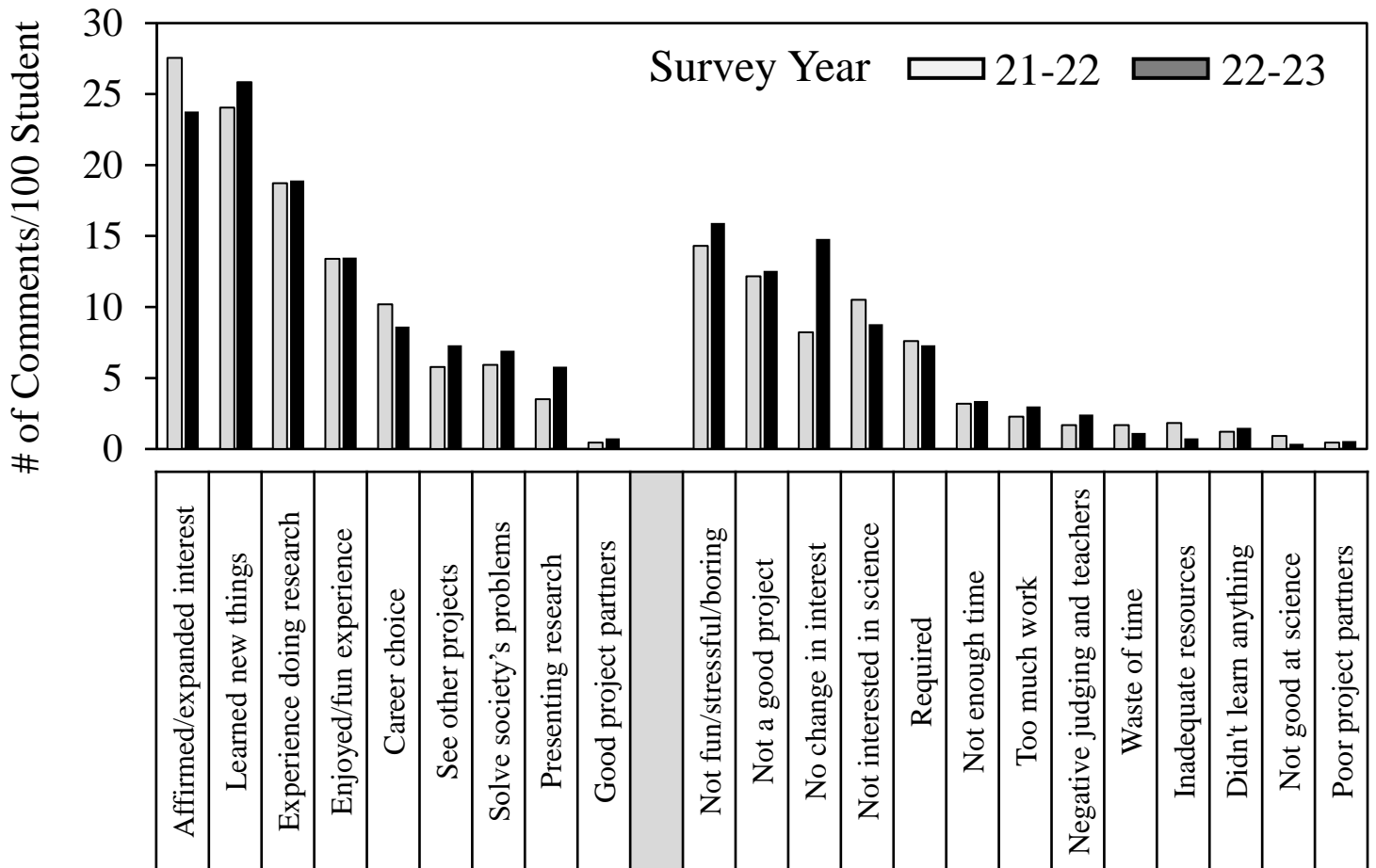

Supplement: S1 Fig — (PDF) [file pone.0325283.s004.pdf]
