# Supplementary material for: High school science fair: What students say—mastery, performance, and self-determination theory
Source: PLoS One. 2025 Jun 25;20(6):e0325283. doi: 10.1371/journal.pone.0325283 (PMC12193632; doi:10.1371/journal.pone.0325283)
Supplement: Attachment — Submitted filename: Response to referees.docx [file pone.0325283.s007.docx]

We appreciated the reviewers’ helpful comments about how to make our paper clearer and have made revisions along the lines that they suggested as summarized below.

Response to Reviewer #1.

1) The Abstract has been revised with the objective of the study clearly stated at the beginning and an explanation of how the findings contribute to existing knowledge at the end.

2) The Introduction has been revised with the research objective and contribution presented earlier and points about benefits of coaching etc. moved to the Results and Discussion sections.

3) Instead of the Materials and Methods, the second paragraph of the Discussion now points out and comments on the various limitations of the study including the 3% response rate and geographic coverage.

4) Chi-square statistics mentioned in Materials and Methods refers to Table 2, which has been moved from Supplemental data to the Results section as requested by Reviewer #2 -- *a summary of the key question categories in the main text (not only the appendix) would aid understanding.*

5) Repetition of certain statistics (comments and percentages) has been maintained in the Discussion so that readers can follow the text without turning back to earlier sections.

6) The Discussion section has been revised to present more clearly the potential for targeted interventions to support underrepresented groups such as Black students.

Response to Reviewer #2.

The concerns of Reviewer #2 overlap closely with those of Reviewer #1 and have been addressed as follows:

1) Clearer statement of the study’s research question and significance of the findings in the revised Abstract and Introduction

2) Clearer statement of research gap the study seeks to address in the revised Abstract and Introduction

3) Data coming from only seven U.S. states and student demographics and SEF contexts within those states and 3% response rate. – New paragraph regarding limitations in the Discussion (2^nd^ paragraph).

4) Summary of key question categories -- now Table 2 (chi square results) -- in the Results and not the Supplemental info.

5) The potential impact of SEF structures developed into concrete recommendations in the revised Discussion
